# Vicarious Menstruation

**Published:** 1917-01

**Authors:** 


### ABSTRACTS

Have you ever felt that this department of the BUFFALO MEDICAL JOURNAL was lacking in abstracts of your specialty, or that such as were published lacked the expert judgement which you yourself could have exercised in selection and preparation? If not, you are more charitable than the editor has been to himself. If so, will you assist in abstracting from a few journals, and thus make this department more representative and more valuable? Incidentally, there are few forms of study more helpful to the worker than this.

It happened that for the December issue there was exactly enough news matter to fill the Journal, without printing any abstracts except those to fill in pages at the end of originals and the advertising pages. It may be frankly admitted that the inclusion of genuine scientific abstracts in the advertising section is partly to insure the reading of this section. That this may not seem too mercenary a motive, it may be stated with equal frankness that this section is what enables a journal to be issued at customary subscription rates instead of a prohibitive amount. Another and more cogent reason is that it is necessary to allow some elasticity in the amount of advertising space, and that the use of scientific abstracts tends to discourage an excess of ''readers.'' After more than five years, we have still not been forced or even permitted to supplant original articles by the issuance of an ''all-abstract'' number, although for many reasons such an issue has seemed desirable to many of our readers. We wish to repeat that the preparation of abstracts is a valuable means of medical study, that the editor does not begrudge the time necessary, but that he feels that a more general co-operation in the preparation of abstracts would give a broader review of medical literature and a higher grade of judgment in selection and comment, than can be secured from a department of this kind mainly due to one source.

**Vicarious Menstruation.** U. G. M. Walker of Flint, Texas, Med. World, Nov., relates the case of a unipara aged 27 who began 3 years previously to have regular menstrual haematemesis in place of the previously normal uterine flow.

